# Supplementary material for: Interactive Responses of Solanum Dulcamara to Drought and Insect Feeding are Herbivore Species-Specific
Source: Int J Mol Sci. 2018 Dec 3;19(12):3845. doi: 10.3390/ijms19123845 (PMC6321310; doi:10.3390/ijms19123845)
Supplement: Supplementary file 1 [file ijms-19-03845-s001.pdf]

Supplementary data

Interactive responses of *Solanum dulcamara* to drought and insect feeding are herbivore species specific

Nguyen et al

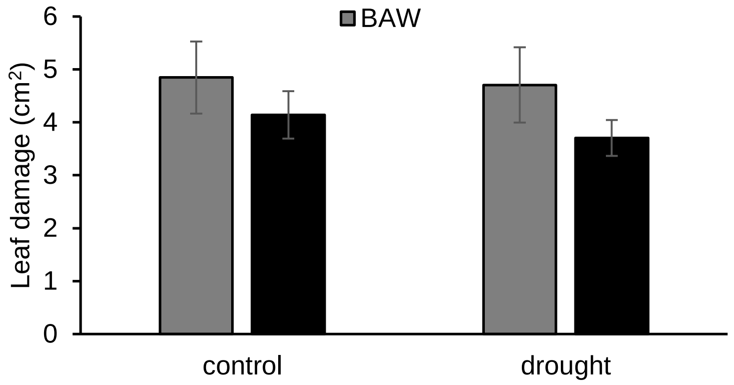

Figure S1. Levels of herbivory damage of leaf samples used for microarray analysis, measurements of hormone concentrations and protease inhibitor contents. *Spodoptera exigua* (BAW, grey bars) and *Leptinotarsa decemlineata* (CPB, black bars) larvae were fed for 48 h on *Solanum dulcamara* plants under well-watering (control) or drought treatments. Error bars are SEM; N = 9 per treatment combination.

Table S1. Univariate test for effects of watering treatments (well-watered and drought) and herbivory treatments (no herbivory or 48-h herbivory by *Spodoptera exigua* or *Leptinotarsa decemlineata*) on concentrations of jasmonic acid (JA), JA-isoleucine (JA-Ile), abscisic acid (ABA) and salicylic acid (SA) in *Solanum dulcamara*. Degree of freedom (df) and *F* values were presented. * $p < 0.05$; ** $p < 0.01$; *** $p < 0.001$. Bold numbers indicate significance.

Source	df	JA		JA-Ile		ABA		SA	
		F		*F*		*F*		*F*	
Water	1	**4.1**	*	2.2		**58.0**	***	0	
Herbivory	2	**101.5**	***	**96.0**	***	**10.6**	***	**12.4**	***
Water * Herbivory	2	**3.5**	*	**4.6**	*	**6.7**	**	1.5	

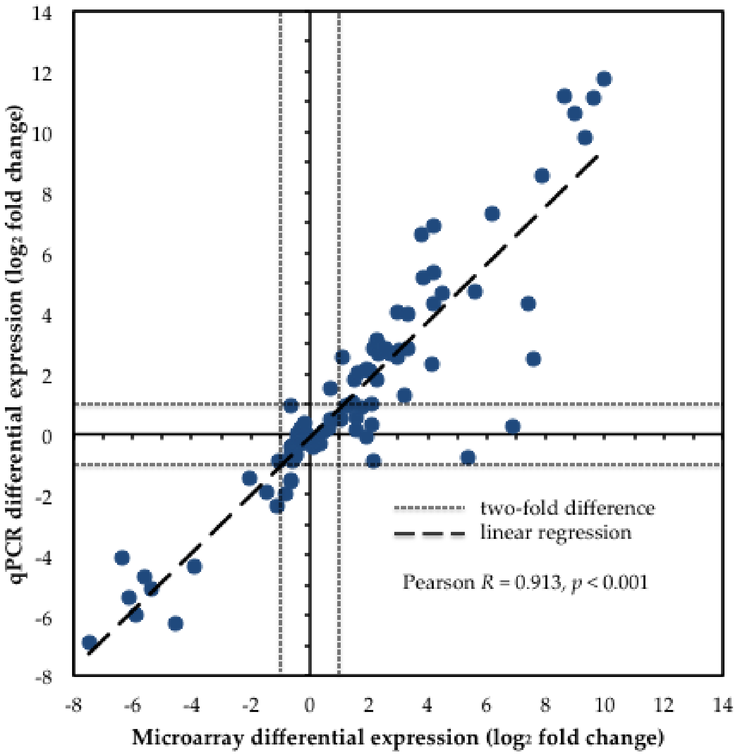

Figure S2. Quantitative PCR validation of microarray analysis of differential gene expression. Correlation plot between log2 values of expression fold changes of 16 target and four reference genes. Each dot is a comparison between a treatment combination (water availability x herbivory treatments) to the control (well-watering conditions without herbivory).

Table S2. Overrepresentation of gene ontology (GO) terms among genes that were always induced by insect herbivory on *Solanum dulcamara* regardless of watering treatments (part i in Figure 4C). Fisher's exact tests were used with FDR *q* value cut-off at 0.05. GO categories (Cat.): Molecular Function (F), Biological Process (P), Cellular Component (C).

GO-ID	Term description	Cat.	*q* value	#Gene
15 top GO terms enriched in upregulated genes				
GO:0004866	endopeptidase inhibitor activity	F	1.23E-08	10
GO:0009505	plant-type cell wall	C	1.81E-05	24
GO:0009611	response to wounding	P	8.29E-05	32
GO:0048046	apoplast	C	1.11E-04	24
GO:0009695	jasmonic acid biosynthetic process	P	1.49E-04	15
GO:0009605	response to external stimulus	P	3.27E-04	73
GO:0019373	epoxygenase P450 pathway	P	4.11E-04	3
GO:0047987	hydroperoxide dehydratase activity	F	4.11E-04	3
GO:0009269	response to desiccation	P	9.42E-04	9
GO:0009753	response to jasmonic acid	P	1.47E-03	28
GO:0009698	phenylpropanoid metabolic process	P	2.70E-03	18
GO:0009978	allene oxide synthase activity	F	2.71E-03	3
GO:0044550	secondary metabolite biosynthetic process	P	7.18E-03	22
GO:0000326	protein storage vacuole	C	7.20E-03	5
GO:0042807	central vacuole	C	8.95E-03	4
15 top GO terms enriched in downregulated genes				
GO:0009644	response to high light intensity	P	8.46E-31	35
GO:0042542	response to hydrogen peroxide	P	5.47E-30	34
GO:0010286	heat acclimation	P	1.69E-16	20
GO:0051082	unfolded protein binding	F	7.61E-16	17
GO:0034976	response to endoplasmic reticulum stress	P	4.52E-14	27
GO:0042026	protein refolding	P	9.17E-08	7
GO:0001671	ATPase activator activity	F	1.34E-07	5
GO:0032781	positive regulation of ATPase activity	P	1.34E-07	5
GO:0006986	response to unfolded protein	P	4.81E-07	16
GO:0031072	heat shock protein binding	F	5.32E-07	10
GO:0051087	chaperone binding	F	1.06E-06	7
GO:0045109	intermediate filament organization	P	3.39E-06	4
GO:0005730	nucleolus	C	2.36E-05	24
GO:0005783	endoplasmic reticulum	C	4.49E-05	21
GO:0005782	peroxisomal matrix	C	1.09E-04	5

Table S3. Univariate test for effects of watering treatments (well-watered and drought) and herbivory treatments (no herbivory or 48-h herbivory by *Spodoptera exigua* or *Leptinotarsa decemlineata*) on serine-type protease inhibitor (serPI) and leaf total protein contents in *Solanum dulcamara*. Degrees of freedom (df) and *F* values are presented. * $p < 0.05$; *** $p < 0.001$. Bold numbers indicate significance.

Source	df	serPI		Total protein	
		F		*F*	
Water	1	**73.315**	***	**5.605**	*
Herbivory	2	**4.517**	*	0.158	
Water * Herbivory	2	1.137		0.499	

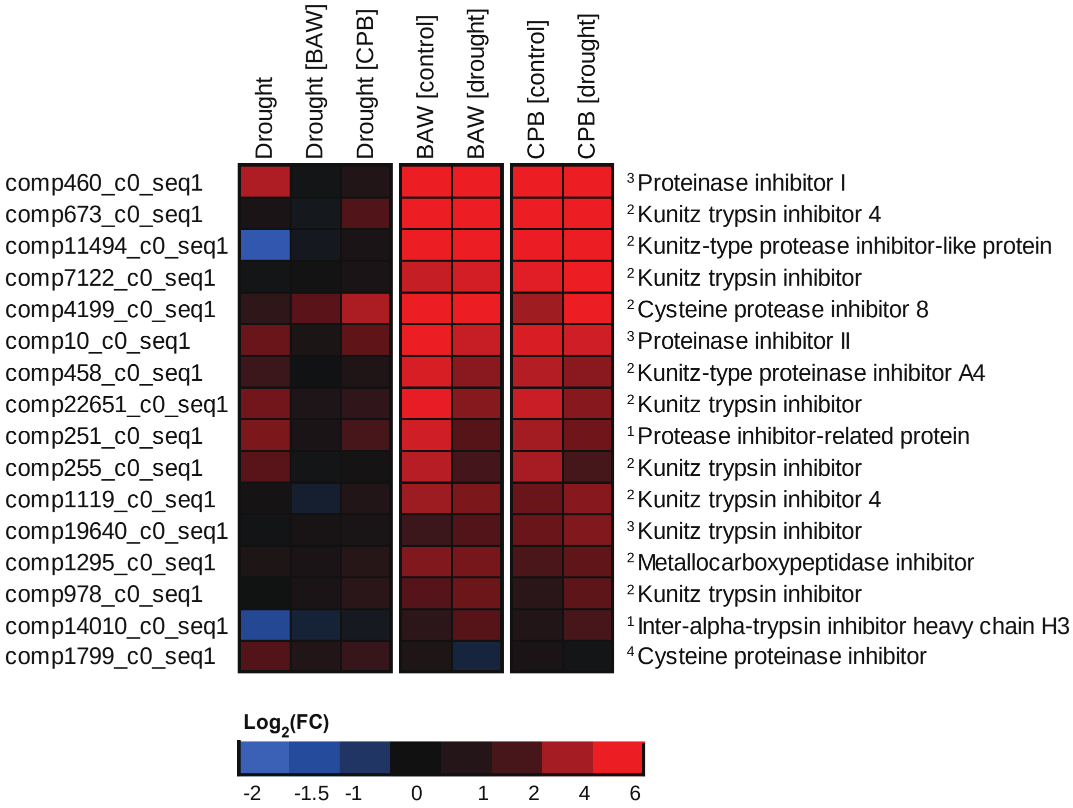

Figure S3. Effects of watering and insect herbivory treatments on the expression of protease inhibitor (PI) genes in *Solanum dulcamara*. Each column is a pairwise comparison between two conditions to show effects of drought treatment on plants that were undamaged (Drought) or fed by *Spodoptera exigua* (Drought [BAW]) or *Leptinotarsa decemlineata* (Drought [CPB]) and effects of herbivory on well-watered (BAW [control] and CPB [control]) or drought-stressed plants (BAW [drought] and CPB [drought]). [1] GO:0030414, peptidase inhibitor activity; [2] GO:0004866, endopeptidase inhibitor activity [3] GO:0004867, serine-type endopeptidase inhibitor activity [4] GO:000486, cysteine-type endopeptidase inhibitor activity.

(A)

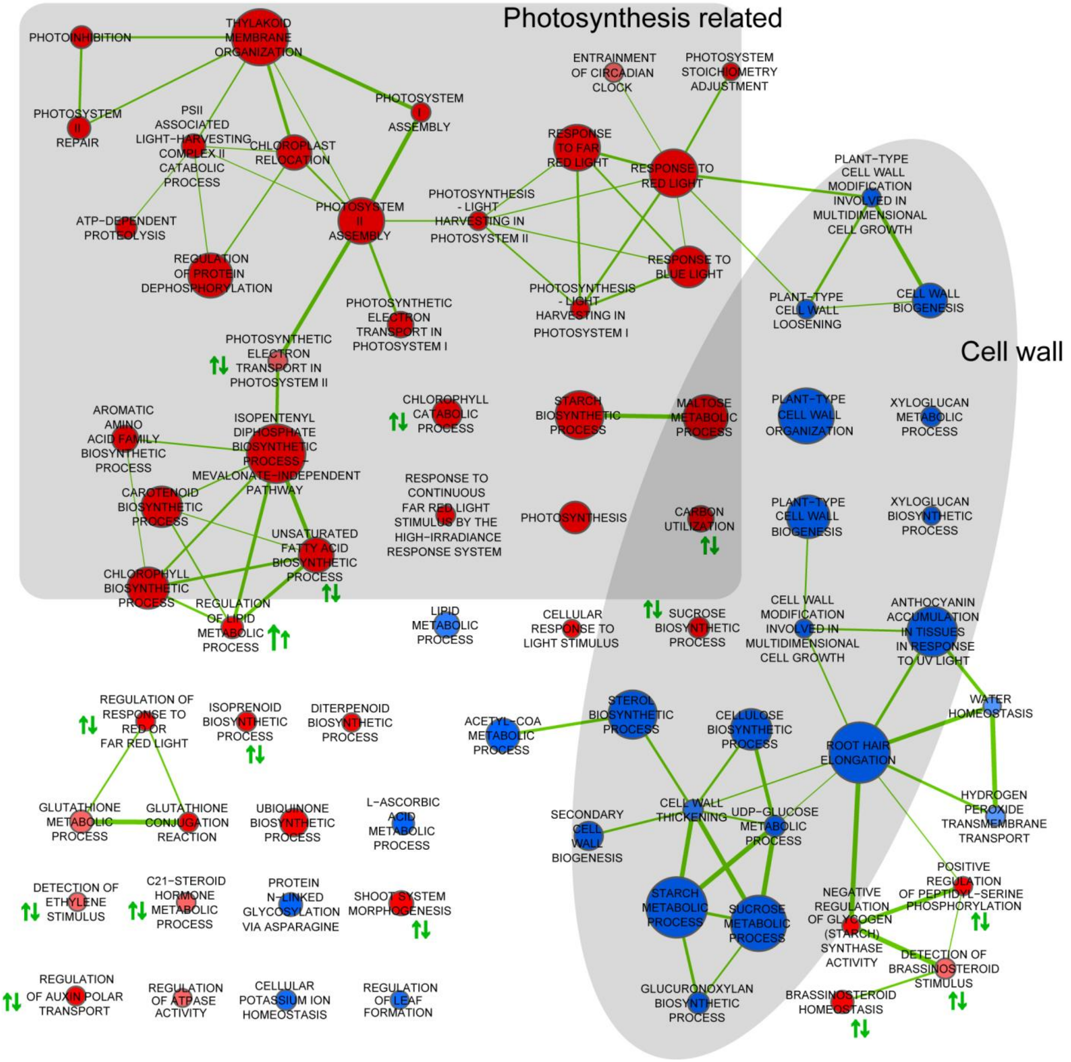

(Figure S4. Continued)

(B)

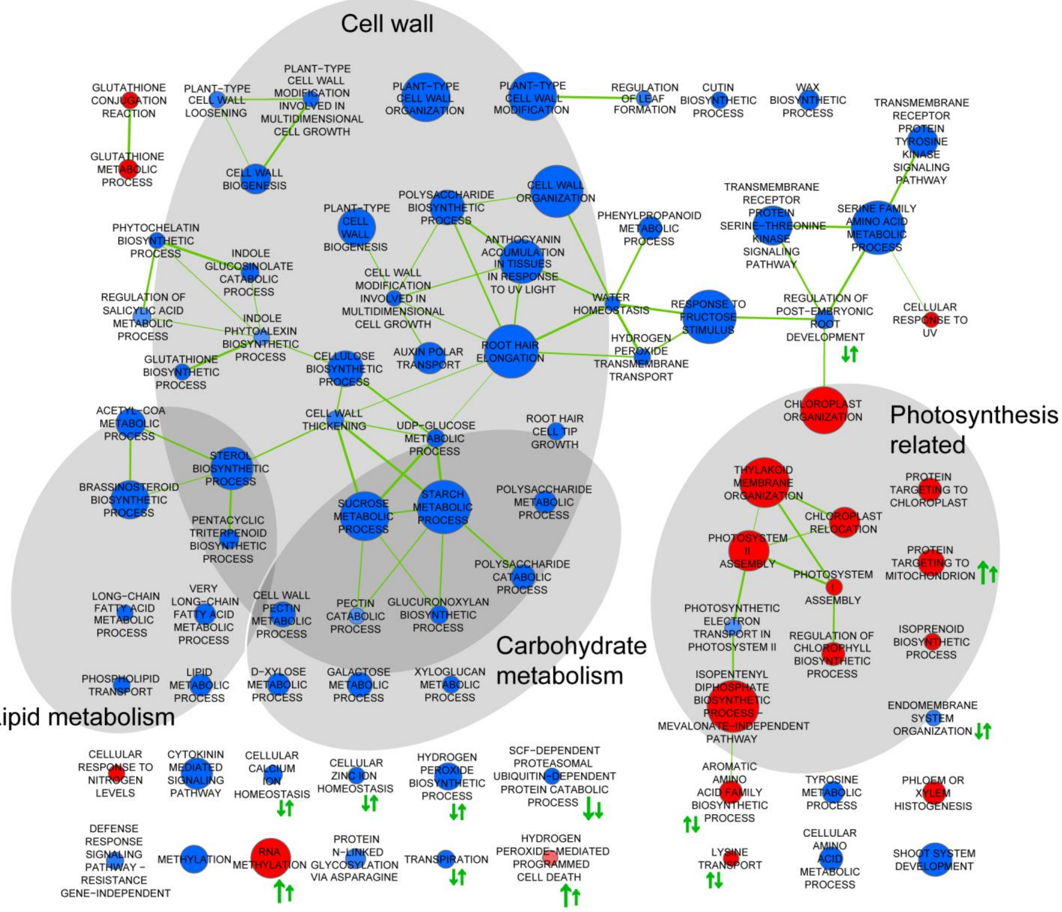

Figure S4. Differences in the transcriptional regulation *Solanum dulcamara* plants subjected to herbivory by *Spodoptera exigua* (BAW) or *Leptinotarsa decemlineata* (CPB). Network of biological processes (BPs), indicated by nodes, in well-watered (**A**) or drought-stressed (**B**) plants. Only BPs with a nominal *p* value ≤ 0.001, FDR *q* value ≤ 0.05, and an overlap coefficient ≤ 0.5 generated from the gene set enrichment analyses were included in the networks. Red or blue nodes are BPs that were downregulated or upregulated, respectively, stronger by BAW than by CPB compared to undamaged plants. Exceptions to this colour coding are nodes with adjacent green arrows indicating inducing directions by CPB (left arrow) and BAW (right arrow) herbivory. Node and edge sizes correspond to numbers of genes of a BP and number of genes that overlap between two connected BPs, respectively. Related clusters and individual BPs are grouped and assigned with labels.

Table S4A. Overrepresentation of gene ontology (GO) terms among the genes that were specifically induced by *Spodoptera exigua* (BAW) herbivory on *Solanum dulcamara* plants under well-watering or drought treatments (parts o + k + g in Figure 4C). Fisher's exact tests were used with FDR *q* value cut-off at 0.05. GO categories (Cat.): Molecular Function (F), Biological Process (P) and Cellular Component (C).

GO-ID	Term description	Cat.	*q* value	# Gene
GO terms enriched in BAW-upregulated genes specific to control conditions				
GO:0009607	response to biotic stimulus	P	3.46E-03	71
GO:0030397	membrane disassembly	P	3.46E-03	4
GO:0009414	response to water deprivation	P	3.46E-03	35
GO:0051707	response to other organism	P	3.46E-03	69
GO:0005976	polysaccharide metabolic process	P	3.46E-03	44
GO:0005985	sucrose metabolic process	P	5.49E-03	17
GO:0009505	plant-type cell wall	C	1.66E-02	21
GO:0048509	regulation of meristem development	P	2.16E-02	22
GO:0016165	linoleate 13S-lipoxygenase activity	F	2.16E-02	4
GO:0005576	extracellular region	C	2.79E-02	29
GO:0009695	jasmonic acid biosynthetic process	P	2.97E-02	13
GO:0071669	plant-type cell wall organization or biogenesis	P	2.97E-02	30
GO:0071555	cell wall organization	P	4.81E-02	33
15 top GO terms enriched in BAW-upregulated genes overlapping between control and drought treatments				
GO:0045229	external encapsulating structure organization	P	2.06E-06	45
GO:0009505	plant-type cell wall	C	2.06E-06	27
GO:0042546	cell wall biogenesis	P	1.69E-05	29
GO:0010054	trichoblast differentiation	P	3.83E-05	30
GO:0051753	mannan synthase activity	F	4.74E-05	5
GO:0009506	plasmodesma	C	4.90E-05	55
GO:0005886	plasma membrane	C	1.81E-04	80
GO:0030244	cellulose biosynthetic process	P	2.19E-04	13
GO:0016760	cellulose synthase (UDP-forming) activity	F	3.54E-04	6
GO:0005985	sucrose metabolic process	P	4.27E-04	17
GO:0006011	UDP-glucose metabolic process	P	8.64E-04	6
GO:0048046	apoplast	C	8.66E-04	23
GO:0005802	trans-Golgi network	C	1.03E-03	13
GO:0052546	cell wall pectin metabolic process	P	1.73E-03	10
GO:0042545	cell wall modification	P	1.97E-03	22
GO terms enriched in BAW-upregulated genes specific to drought treatment				
GO:0005783	endoplasmic reticulum	C	2.94E-02	44
GO terms enriched in BAW-downregulated genes specific to control conditions				
GO:0016556	mRNA modification	P	1.59E-03	13
GO:0042793	transcription from plastid promoter	P	1.51E-02	8
GO:0009507	chloroplast	C	4.73E-02	61
GO:0009902	chloroplast relocation	P	4.73E-02	10
GO:0019288	isopentenyl diphosphate biosynthetic process, non-MVA pathway	P	4.73E-02	13
GO:0006098	pentose-phosphate shunt	P	4.73E-02	12
GO:1903792	negative regulation of anion transport	P	4.73E-02	4

GO:0010362	negative regulation of anion channel activity by blue light	P	4.73E-02	4
GO terms enriched in BAW-downregulated genes overlapping between control and drought treatments				
GO:0016556	mRNA modification	P	2.03E-03	9
GO:0044434	chloroplast part	C	5.97E-03	26
GO:0019684	photosynthesis, light reaction	P	6.37E-03	12
GO:0000959	mitochondrial RNA metabolic process	P	4.20E-02	5
GO:0031425	chloroplast RNA processing	P	4.20E-02	5
GO:0009311	oligosaccharide metabolic process	P	4.86E-02	12
GO terms enriched in BAW-downregulated genes specific to drought treatment				

Table S4B. Overrepresentation of gene ontology (GO) terms among the genes that were specifically induced by *Leptinotarsa decemlineata* (CPB) herbivory on *Solanum dulcamara* plants under well-watering (control) or drought treatments (parts a + b + c in Figure 4C). Fisher's exact tests were used with FDR *q* value cut-off at 0.05. GO categories (Cat.): Molecular Function (F), Biological Process (P) and Cellular Component (C).

GO-ID	Term description	Cat.	*q* value	# Gene
GO terms enriched in CPB-upregulated genes specific to control conditions				
GO terms enriched in CPB-upregulated genes overlapping between control and drought treatments				
GO terms enriched in CPB-upregulated genes specific to drought treatment				
GO:0006614	SRP-dependent cotranslational protein targeting to membrane	P	5.43E-28	25
GO:0006415	translational termination	P	9.99E-28	25
GO:0019083	viral transcription	P	1.07E-27	25
GO:0006414	translational elongation	P	5.17E-27	25
GO:0000184	nuclear-transcribed mRNA catabolic process, nonsense-mediated decay	P	2.23E-26	25
GO:0044391	ribosomal subunit	C	3.67E-25	27
GO:0003735	structural constituent of ribosome	F	3.33E-24	27
GO:0006413	translational initiation	P	4.03E-23	25
GO:0022625	cytosolic large ribosomal subunit	C	1.31E-22	21
GO:0001510	RNA methylation	P	8.32E-19	22
GO:0003723	RNA binding	F	2.79E-05	20
GO:0022627	cytosolic small ribosomal subunit	C	3.82E-04	6
GO:0005730	nucleolus	C	5.17E-04	22
GO:0005794	Golgi apparatus	C	9.12E-04	25
GO:0015808	L-alanine transport	P	2.49E-03	2
GO:0015180	L-alanine transmembrane transporter activity	F	2.49E-03	2
GO:0015812	gamma-aminobutyric acid transport	P	7.04E-03	2
GO:0015185	gamma-aminobutyric acid transmembrane transporter activity	F	7.04E-03	2
GO:0000028	ribosomal small subunit assembly	P	8.72E-03	3
GO:0016020	membrane	C	3.19E-02	51
GO terms enriched in CPB-downregulated genes specific to control conditions				
GO terms enriched in CPB-downregulated genes overlapping between control and drought treatments				
GO terms enriched in CPB-downregulated genes specific to drought treatment				
GO:0010378	temperature compensation of circadian clock	P	1.01E-03	3
GO:0048578	positive regulation of long-day photoperiodism, flowering	P	1.05E-02	3

Table S5. The top 10 genes specifically induced by *Spodoptera exigua* (BAW) or *Leptinotarsa decemlineata* (CPB) in drought-stressed *Solanum dulcamara* plants.

Gene number	Description
10 genes most upregulated by BAW specifically under drought; in part g, Figure 4C	
comp4199_c0_seq1	Cysteine protease inhibitor 8
comp13011_c0_seq2	Sesquiterpene synthase 1
comp29348_c0_seq1	Lipid transfer protein
comp26311_c0_seq1	ABC transporter G family member 14
comp26586_c0_seq1	Limonene synthase
comp25136_c0_seq1	Legumin 11S-globulin
comp5470_c0_seq1	Peroxidase
comp11400_c0_seq1	Multidrug resistance protein mdtK
comp13940_c0_seq1	Pectate lyase
comp11103_c0_seq1	DNA polymerase
10 genes most downregulated by BAW specifically under drought; in part g, Figure 4C	
comp17359_c0_seq1	Protein with unknown function
comp904_c0_seq1	Microtubule-associated protein futsch
comp20050_c0_seq1	Phloem protein
comp22520_c0_seq1	SKP1-like protein
comp12519_c0_seq1	Homeobox-leucine zipper protein
comp13007_c0_seq1	Heat shock protein 1
comp20351_c0_seq1	Cotton fiber expressed protein 1
comp5436_c1_seq1	Early nodulin 75 protein
comp23666_c0_seq1	Acetyl esterase
comp16023_c0_seq1	Protein with unknown function
10 genes most upregulated by CPB specifically under drought; in part c, Figure 4C	
comp15794_c0_seq1	Protein with unknown function
comp572_c0_seq1	Osmotin-like protein
comp15807_c0_seq1	Patatin-like protein 3
comp20396_c0_seq1	Alternative oxidase
comp2990_c0_seq1	Universal stress protein
comp10454_c0_seq1	Tropinone reductase I
comp4593_c0_seq1	N-acetyltransferase
comp469_c0_seq1	Blue copper protein
comp19640_c0_seq1	Kunitz trypsin inhibitor
comp4880_c0_seq1	Protein with unknown function
10 genes most downregulated by CPB specifically under drought; in part c, Figure 4C	
comp21048_c0_seq1	Protein with unknown function
comp24351_c0_seq1	Protein with unknown function
comp23146_c0_seq1	Carotenoid cleavage dioxygenase
comp2675_c0_seq2	Squamosa promoter binding-like protein
comp19061_c0_seq1	BZIP transcription factor family protein
comp1493_c0_seq1	Pathogenesis-related protein PR-1
comp25443_c0_seq1	Protein with unknown function
comp21508_c0_seq1	Protein with unknown function
comp28418_c0_seq1	Non-LTR retroelement reverse transcriptase
comp959_c0_seq1	Polygalacturonase

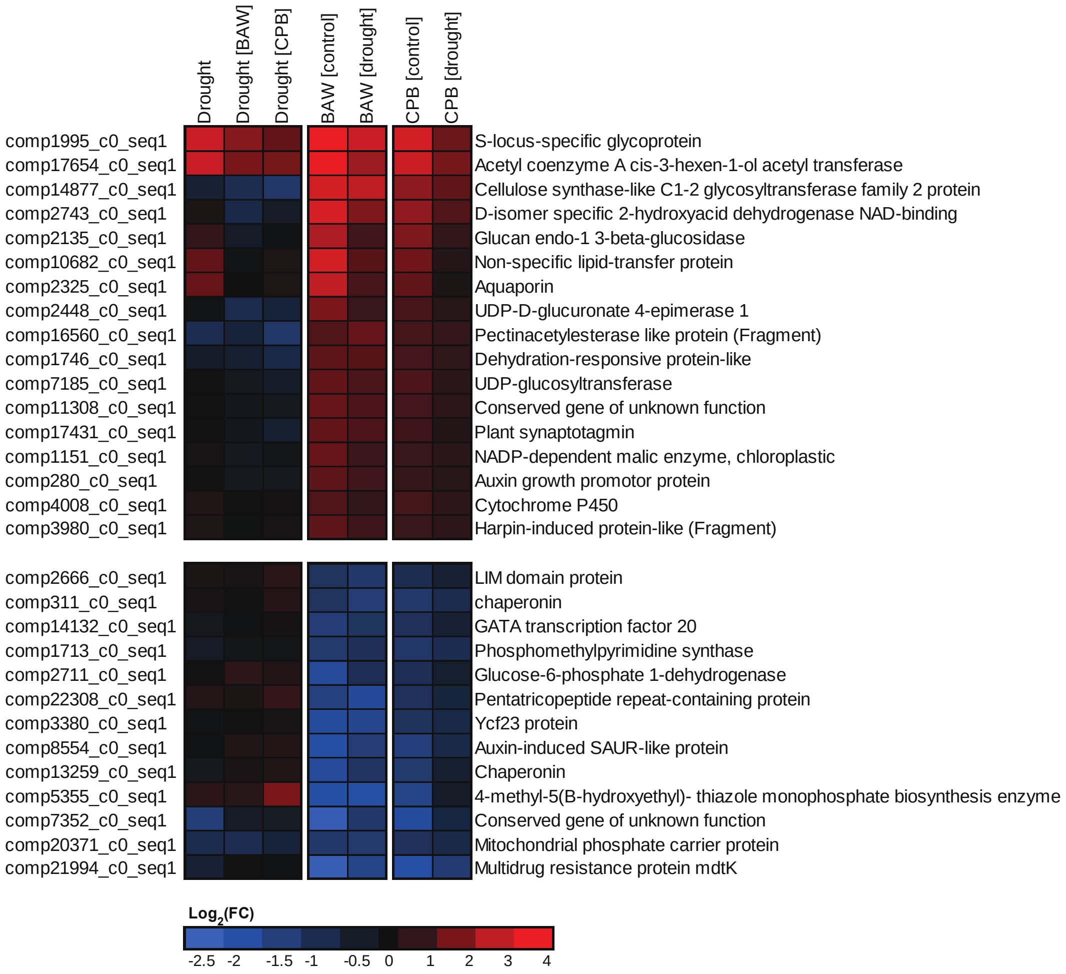

Figure S5. Herbivore-induced genes in *Solanum dulcamara* that were no longer significantly induced by *Leptinotarsa decemlineata* (CPB) feeding on drought-stressed plants. Each column is a pairwise comparison between two conditions to show effects of drought on plants that were undamaged (drought) or fed by *Spodoptera exigua* (drought [BAW]) or *L. decemlineata* (drought [CPB]) and effects of herbivory on well-watered (BAW [control] and CPB [control]) or drought-stressed plants (BAW [drought] and CPB [drought]).

Table S6. Overrepresentation of gene ontology (GO) terms among the genes that were consistently induced by drought and specifically in a 2x2 treatment combinations with *Leptinotarsa decemlineata* herbivory on *Solanum dulcamara* plants. Fisher's exact tests were used with FDR *q* value cut-off at 0.05. GO categories (Cat.): Molecular Function (F), Biological Process (P) and Cellular Component (C).

GO-ID	Term description	Cat.	q value	# Gene
GO terms enriched in upregulated genes				
GO:0009570	chloroplast stroma	C	7.38E-07	24
GO:0019288	isopentenyl diphosphate biosynthetic process, methylerythritol 4-phosphate pathway	P	9.06E-04	11
GO:0009941	chloroplast envelope	C	1.10E-03	20
GO:0009535	chloroplast thylakoid membrane	C	1.43E-03	13
GO:0009657	plastid organization	P	1.20E-02	15
GO:0009767	photosynthetic electron transport chain	P	2.80E-02	6
GO:0015995	chlorophyll biosynthetic process	P	3.44E-02	8
GO:0043229	intracellular organelle	C	3.64E-02	65
GO:0009234	menaquinone biosynthetic process	P	3.64E-02	2
GO:0042254	ribosome biogenesis	P	4.35E-02	12
GO:0044422	organelle part	C	4.53E-02	47
GO terms enriched in downregulated genes				
GO:1901701	cellular response to oxygen-containing compounds	P	7.79E-05	22
GO:0071229	cellular response to acid chemical	P	1.13E-04	19
GO:0080181	lateral root branching	P	1.56E-04	3
GO:0015398	high-affinity secondary active ammonium transmembrane transporter activity	F	2.59E-04	3
GO:0010311	lateral root formation	P	2.93E-03	5
GO:0009751	response to salicylic acid	P	2.93E-03	13
GO:0072488	ammonium transmembrane transport	P	3.73E-03	3
GO:0015843	methylammonium transport	P	4.71E-03	3
GO:0098542	defence response to other organism	P	9.22E-03	18
GO:0001666	response to hypoxia	P	2.61E-02	6
GO:0031347	regulation of defence response	P	3.20E-02	13
GO:0009954	proximal/distal pattern formation	P	3.28E-02	3
GO:0033554	cellular response to stress	P	3.46E-02	20
GO:0009737	response to abscisic acid	P	3.54E-02	14
GO:0009753	response to jasmonic acid	P	4.03E-02	11
GO:0043647	inositol phosphate metabolic process	P	4.06E-02	4
GO:0009755	hormone-mediated signalling pathway	P	4.07E-02	15
GO:0009627	systemic acquired resistance	P	4.14E-02	10
GO:0009605	response to external stimulus	P	4.15E-02	24
GO:0080183	response to photooxidative stress	P	4.93E-02	2

Table S7. Primer sequences used for quantitative validation of microarray analysis.

Gene name	Primer name	Sequence (5'-3')
**Target genes**		
comp14877_c0_seq1	comp14877F	CTGGCGCGTACCTCTTCTTCTG
	comp14877R	TTGGGCATGAAAGTTCCAACGT
comp10_c0_seq1	PIN_20346F	AGCGCTGATGGAACTTTCATTT
	PIN_20346R	CATCAATGGAATCATCATTGTGC
comp15183_c0_seq1	comp15183F	GACGAACACGGTAAACGAGGAG
	comp15183R	GGGCAATACTTCATCCAACGAG
comp17670_c0_seq1	comp17670F	TCAGTGGTGGGATTTCTGGTCT
	comp17670R	TACAAGAGACGTCGGCATCTGA
comp2069_c0_seq1	comp2069F	GCACCAGAGCCATTAGCAATCT
	comp2069R	GGATTCAAGTCTCCCATTCACG
comp15724_c0_seq1	comp15724F	CCTGGCATGTCAAACCTCATTT
	comp15724R	AACGGAGGATGTTGGGTACAGA
comp702_c0_seq1	comp702F	AAACTCTCCGCACATCTCGAAC
	comp702R	GTTCTGGGCTGCTCGTAACATT
comp955_c0_seq1	comp955F	GCATAGTCCAGCCGTTATTCCA
	comp955R	GGAAGCCCTGCTTATCTTCGAT
comp28407_c0_seq1	comp28407F	TCCAACTCTCGCAATACGTTCA
	comp28407R	GGCATATGGAATGGAAGTCGAG
comp2135_c0_seq1	comp2135F	AAATCCTCTTGCTTCGGGTTGT
	comp2135R	CCCGGTTGGGATGTAGGTATTT
comp572_c0_seq1	comp572F	GAACTGGTCCAAGGCATATTCG
	comp572R	TGCTGGTAGAGGTTCGTGTCAG
comp15807_c0_seq1	comp15807F	ATTCGGAGCTTCCTTTCCTCTG
	comp15807R	AGAAACAGGCCGATACGAACAA
comp13011_c0_seq2	comp13011F	ATATGGAGCGTCGAAGCAAGAG
	comp13011R	GACCTCGTTCGAGAGCAAACAT
comp2617_c0_seq1	comp2617F	TGATTGCTCATCTTGCGTCATT
	comp2617R	AAGGTCCAGGGAACAACTCTCC
comp3079_c0_seq1	comp3079F	TGGACAAGTCATGCACACTCCT
	comp3079R	AGCTGGAAGTTGAACCTGACGA
comp1495_c0_seq1	comp1495F	GGTTTCTCGAATCTCAGGCAGA
	comp1495R	GGTGGGAAAGGAAAGCAAATCT
**Reference genes**		
comp28_c0_seq4	EF1a_48212F	TGGTACCTCCCAGGCTGACTGTG
	EF1a_48212R	GCAACGCATGTTCACGGGTCT
comp3969_c0_seq2	CAC_00903F	GGTAGTGTGCTCCGTTGCGATG
	CAC_00903R	GCGGGATTTAAGCTGCGACTCT
comp10185_c0_seq4	TIP_26454F	CCTTTGCAACAGGTGGTGCTCG
	TIP_26454R	CCCACTGAATGCAGCTGCTTCC
comp141_c0_seq1	GAPDH_13565F	ATTGGTGGCTCGGGTTGCTCTC
	GAPDH_13565R	ATGATGCTTCCACTGGCCGTGT
